# *Cupriavidus* in the intestinal microbiota of Tibet endemic fish *Glyptosternum maculatum* can help it adapt to habitat of the Qinghai Tibet Plateau

**DOI:** 10.1186/s12917-021-03092-5

**Published:** 2021-12-07

**Authors:** Yingzi Pan, Zhichao Li, Jianshe Zhou, Qielu Wang, Huifen Xu, Zhenbo Mou

**Affiliations:** 1 Tibet Academy of Agricultural and Animal Husbandry Sciences, Institute of Fishery Sciences, Lhasa, 850032 China; 2 College of Animal Science and Technology, Henan Agricultural University, Zhengzhou, 450046 China

**Keywords:** *Glyptosternum maculatum*, Intestinal microbiota, *Cupriavidus*, Copper

## Abstract

**Background:**

Gut microbes play an important role in the growth and development of fish. The Tibetan Plateau fish *Glyptosternum maculatum* is a unique species of sisorid catfish living in the river up to 4200 m altitude.

**Results:**

To understand the mechanisms underlying the ability of *G. maculatum* to adapt to the high-altitude habitat, the intestinal microbiota of *G. maculatum* was studied. We used high-throughput sequencing of the 16S ribosomal RNA gene of intestinal microorganisms of wild and cultured *G. maculatum* to explore the characteristics of intestinal microorganisms and compared the gut microbial community of wild and cultured *G. maculatum*. The results showed that the α-diversity and richness of the intestinal microbiome were higher in wild *G. maculatum* than in cultured fish. The most abundant phylum in both *G. maculatum* were *Fusobacteria*, *Proteobacteria*, *Firmicutes*, and *Bacteroidetes*; *Cetobacterium* and *Cupriavidus* are the most dominant genus. The membership and structure of intestinal bacterial communities in wild *G. maculatum* are similar to the cultured fish, suggesting that a core microbiota is present in both *G. maculatum* intestinal bacterial communities. Metastats analysis showed that six genera were differentially represented between the wild and cultured *G. maculatum*.

**Conclusions:**

The most interesting characteristic of the intestinal microbial communities of *G. maculatum* is that there were large numbers of *Cupriavidus*, which may play an important role in the adaptation of *G. maculatum* to the water of the Yarlung Zangbo River with a high Cu content. This result, in turn, can guide us on breeding *G. maculatum*.

## Introduction

The Tibetan Plateau fish *Glyptosternum maculatum* [1] is a sisorid catfish of Siluriformes belonging to Glyptosternoids and is distributed in the Yarlung Zangbo River and its tributaries [2]. *Glyptosternum maculatum* is an omnivorous fish with fish and benthos as the main food source. *Glyptosternum maculatum* is the sole species of sisorid catfish living in the river up to 4200 m altitude [3] and has become an important species in the study of evolution and phylogeny of Sisoridae. However, the development and evolution of *G. maculatum* were affected by interval uplift of the Tibetan Plateau [4] to adapt to the environment. Therefore, the fish provide an excellent resource with which to infer the geological and environmental history of the region [5].

*G. maculatum* is an important commercial fish species in Tibet, and the high demand has caused overexploitation of the natural populations. Consequently, the number of wild *G. maculatum* has decreased sharply, which may eventually lead to the extinction of *G. maculatum*. The protection of fish resources is facing severe challenges. Cultivating *G. maculatum* artificially is an alternative way of meeting the demand for *G. maculatum* without affecting the population of wild fish. Thus, *G. maculatum* has become the major aquaculture fish in Tibet. With the development of aquaculture in Tibet, as well as the important economic value of *G. maculatum*, the potential for controlled rearing of *G. maculatum* is becoming more and more optimistic. However, there are still some problems to be solved in the artificial culture of *G. maculatum*. For example, compared with the wild fish, the cultured *G. maculatum* is more likely to be infected and die.

The gut microbiota occupying the gastrointestinal tract of vertebrates is well known to live in symbiotic association with the vertebrate host, presents a broad range of metabolic activities, and may contribute to various metabolic processes of the host [6]. Thus, microbiota may secrete extracellular enzymes, help the host to complement digestive processes, provide vitamins and enhance nutrition. The microbiota also prevents pathogens from colonizing and competing for nutrients. In addition, they may produce antimicrobial substances and may also modulate the immune system of the host [7–10].

Current information on the intestinal microbiota of fish has largely been derived from culture-based approaches, which often reveal only a limited range of microbial diversity [11]. Comparing the intestinal microflora of cultured *G. maculatum* with the intestinal microflora of wild fish, we can fully understand the intestinal microbiota structure of fish, and how ecological and environmental factors impact fish gut microbiota composition, as well as the role of some important gut microbiota compositions in the growth and development of *G. maculatum*, which is conducive to guiding cultivation of healthy fish to increase the output of fish, and meet the demand of market for *G. maculatum*. The purpose of this study is to compare the intestinal microbiological differences between wild and cultured *G. maculatum* to guide the rearing of *G. maculatum*.

## Materials and methods

### Experimental fish and sampling procedures

The study was carried out in compliance with the ARRIVE guidelines. Five wild *G. maculatum* collected randomly from Xie Tongmen River (group A1–5) and Five cultured *G. maculatum* collected randomly from the Tibet Academy of Agricultural and Animal Husbandry Sciences, Institute of Fishery Sciences (group B1–5) (Table 1). Due to the restriction of growth conditions, the weight of the wild fish of the same age is about two times less than the weight of the cultured fish. The fish showed no visual signs of disease and remained healthy throughout the study. The fish were aseptically captured and immediately kept cold at 4 °C with ice bags during transport to the laboratory. The body weight and body length of the fish were measured, and then the belly of the fish was washed with 75% ethanol. Finally, the intestinal contents of the fish were collected under sterile conditions and then stored at − 80 °C until further processing.

**Table 1:** Summary of total length and body weight of *G. maculatum* samples

Sample	Length(mm)	weight(g)
A1	161	42.5
A2	149	33.3
A3	145	30.6
A4	150	34.0
A5	147	31.9
B1	196	78.8
B2	204	89.4
B3	202	86.7
B4	199	82.7
B5	189	70.3

Note: A1–5, wild *G. maculatum* samples; B1–5, cultured *G. maculatum* samples

#### Ethical approval

For the welfare of the animals, all experiments and animal procedures were conducted strictly according to the protocols recommended by the Institutional Animal Care and Use Committee (IACUC) of Tibet Academy of Agricultural and Animal Husbandry Sciences (permit number: 21–0354) and the protocols supported by the regulation of experimental animal of Ministry of Science and Technology in China,2014. All experiments and methods were performed to minimize animal suffering.

### Intestinal DNA extraction and 16S rRNA gene sequencing

The PowerFecal® DNA Isolation Kit (MoBio Laboratories, Carlsbad, CA, United States) was then used to isolate DNA of intestinal samples following the manufacturer’s recommendations. The quantities and qualities of DNA extracted were checked using a NanoDrop 2000 (ThermoFisher, Wilmington, DE, United States) and gel electrophoresis. Then submitted DNA samples to the Shanghai Personal Biotechnology Co., Ltd., for 16S rRNA gene sequencing. PCR amplification of the bacterial 16S rRNA gene V3-V4 region was performed using the forward primer (5′-ACTCCTACGGGAGGCAGCA-3′) and the reverse primer (5′-GGACTACHVGGGTWTCTAAT-3′). High throughput sequencing procedures refer to Wang et al. [12].

### Bioinformatics analysis

Sequencing reads of the 16S rRNA gene were spliced with the barcode and primer sequence and merged using FLASH software [13]. The quality of the reads was assessed using QIIME software [14] to obtain the high-quality clean tags. The tags were compared with the reference database using the UCHIME algorithm [15] to detect and remove chimera sequences. The reads were then clustered into operational taxonomic units (OTUs) based on an identity threshold of 97% using UPARSE [16]. Taxonomy assignment of the OTUs was performed by comparing sequences with the GreenGene Database based on Ribosomal Database Project (RDP) classifier algorithm to annotate taxonomic information [17]. Multiple sequence alignment of OTUs was performed by MUSCLE software to analyze the phylogenetic relationship of different OTUs and the differences of dominant species in different samples [18]. Rarefaction curves, alpha diversity and beta diversity were calculated by QIIME software. The unweighted UniFrac phylogenetic distance metric was analyzed by using a Principal Coordinate Analysis (PCoA) and Unweighted Pair Group Method with Arithmetic mean (UPGMA) Clustering. PCoA analysis was displayed by WGCNA package, stat packages and ggplot2 package in R softwar.

### Function prediction of *G. maculatum* internal microbiota

The analysis process of picrust2 is as follows, 1) Firstly, align the 16S rRNA gene sequences of known microbial genomes, construct an evolutionary tree, and infer the gene function spectrum of their common ancestors. 2) The 16S rRNA characteristic sequence was aligned with the reference sequence to construct a new evolutionary tree. 3) Using castor hidden state prediction algorithm, according to the copy number of gene family corresponding to the reference sequence in the evolutionary tree, infer the nearest sequence species of the characteristic sequence, and then obtain the copy number of gene family. 4) Combined with the abundance of characteristic sequences of each sample, the copy number of gene family of each sample was calculated. 5) Finally, the gene family is “mapped” to various databases, and minpath is used by default to infer the existence of metabolic pathways, so as to obtain the abundance data of metabolic pathways in each sample. According to the metabolic pathway database and certain calculation methods, the abundance value of metabolic pathway can be obtained. KEGG database is used in this study. The core of KEGG database is KEGG pathway database, http://www.genome.jp/kegg/pathway.html). Among them, metabolic pathways are divided into six categories, including metabolism, genetic information processing, environmental information processing, cellular processes, organic systems and human diseases. Each category of metabolic pathways is further divided into multiple levels.

### Statistical analysis

A completely randomized test design was used in this study. The significance of the difference between means of the groups was determined by Students *t*-test. *P*-values < 0.05 were considered significant. Statistical calculations used in this study were performed using IBM SPSS (24.0).

## Results

### The operational taxonomic units (OTUs) between wild and cultured *G. maculatum*

**Fig. 1:**
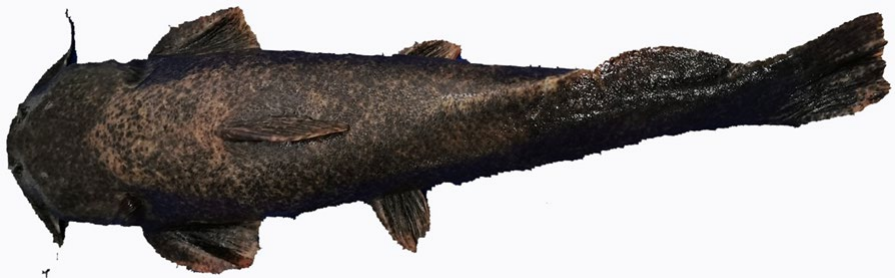
Venn diagram of the two groups showing the quantity variance of OTUs (**A** wild *G. maculatum*; **B** cultured *G. maculatum*)

A total of 587,199 high-quality sequences were obtained from 16S rRNA gene sequencing, ranging from 131 to 458 bp, acquired from the 10 samples. Group A (Wild *G. maculatum*) has the largest number of OTUs, 2072, while group B (Cultured *G. maculatum*) has a smaller number of OTUs, 1814. 1406 OTUs are shared by two *G. maculatum* groups, and the number of unique OTUs in group A is the highest, 666, while the number of unique OTUs in group B is 408 (Fig. 1). The OTUs comprised 28 bacterial phyla, 75 classes, 128 orders, 229 families, 380 genera, and 439 species (Table 2).

**Table 2:** The number of OTUs at different annotated taxonomic level

Sample	OTUs	Phylum	Class	Order	Family	Genus	Species
A1	1128	23	45	63	100	110	48
A2	985	19	41	58	81	110	47
A3	924	23	48	63	91	100	43
A4	903	18	42	59	84	97	40
A5	873	21	47	67	96	113	45
B1	813	20	44	62	81	98	39
B2	836	16	38	49	79	93	35
B3	835	19	43	61	83	95	36
B4	850	22	45	58	83	93	32
B5	843	16	38	51	78	100	44
Total	–	28	75	128	229	380	439

Note: A1-A5: wild *G. maculatum*; B1-B5: cultured *G. maculatum*

### Alpha diversity of *G. maculatum* intestinal microbiota

**Fig. 2:**
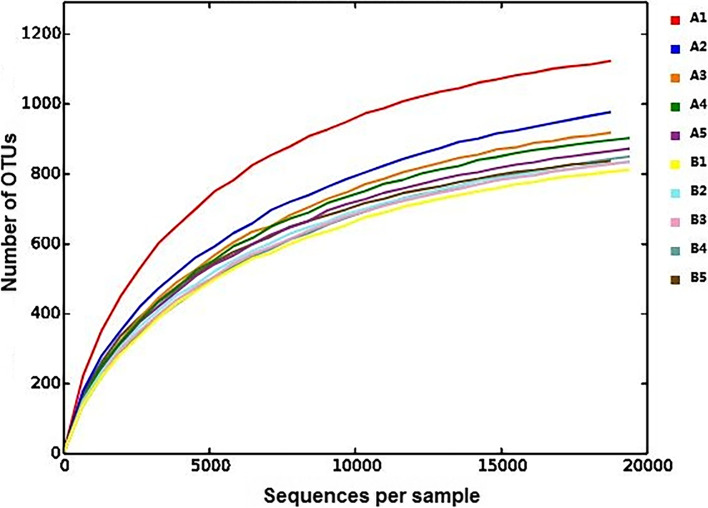
Alpha rarefaction curves of wild and cultured *G. maculatum* gut microbiota (A1-A5: wild *G. maculatum*; B1-B5: cultured *G. maculatum*)

The results of rarefaction curve showed that the curve of each sample tended to flatten with higher sequence numbers, indicating that the sample size was reasonable (Fig. 2). The results of alpha diversity index (Table 3) showed that the indexes of Chao1, ACE and Shannon in group A were significantly higher than those in group B. Although the Simpson and goods coverage indexes in group A were not significantly different from those in group B, they were higher than those in group B. The index showed that intestinal microbiota diversity and relative abundance were higher in wild *G. maculatum* samples (group A) than in cultured *G. maculatum* samples (group B).

**Table 3:** Summary of α-diversity of wild and cultured *G. maculatum* gut microbiota

Alpha diversity index	Group A	Group B	*P*-value
Simpson	0.91232 ± 0.00510	0.89358 ± 0.00158	> 0.05
Chao1	1046.47 ± 20.15	912.88 ± 18.36	*<* 0.05
ACE	1085.14 ± 31.68	945.76 ± 25.85	*<* 0.05
Shannon	5.89 ± 0.15	5.46 ± 0.13	*<* 0.05
Goods coverage	0.99825 ± 0.02082	0.99023 ± 0.00432	> 0.05

Note: *P <* 0.05 means significant difference; *P* > 0.05 means no significant difference

### Differences of *G. maculatum* intestinal microbiota at phylum level

**Fig. 3:**
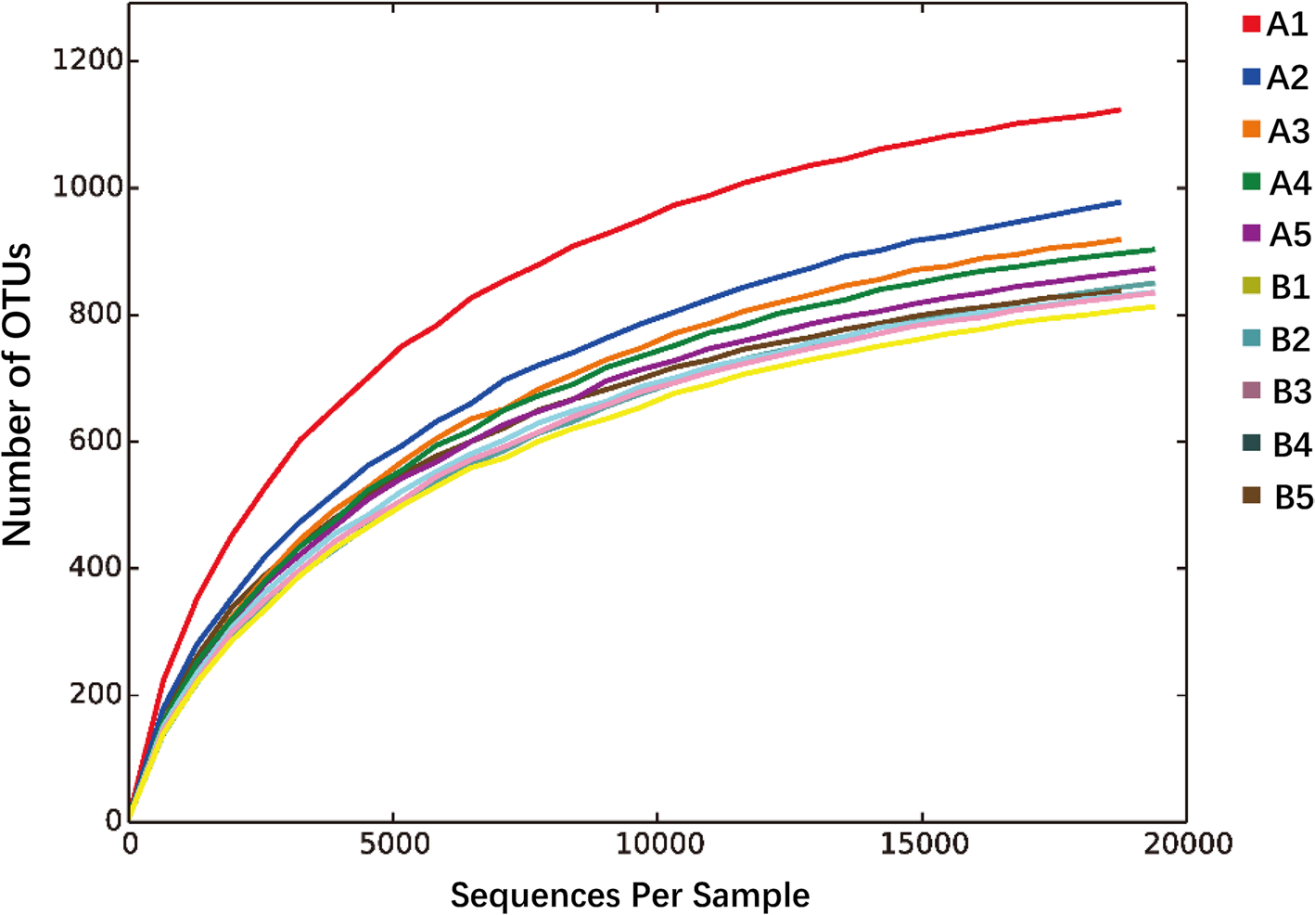
Compositions of *G. maculatum* gut microbiota communities at the phylum and genus levels. Each bar represents the average abundance of each bacterial taxon (different colors) within a group at the phylum level (**a**) and genus level (**b**)

The most abundant taxa of bacteria at the phylum level are shown in Fig. 3a. The most abundant phylum was *Fusobacteria* in all samples, accounting for 30.1 to 42.3% of the total bacterial sequences, indicating that the highest quantity of gut bacterial species are from this taxon. *Proteobacteria* was the second most common phylum, accounting for 22.1 to 44.9%. Other common taxa were *Firmicutes*, *Bacteroidetes*, *Actinobacteria*, and *Chloroflexi*, ranging between 0.5 and 14.5% in all the experimental samples.

### Differences of *G. maculatum* intestinal microbiota at genus level

Composition of microbiota at the genus level is represented by two genera, *Cetobacterium* and *Cupriavidus.* Other major genera (> 1%) include some genera of unclassified *Aeromonadaceae*, *Acinetobacter*, *Anoxybacillus*, *Clostridium*, *Ochrobactrum*, unclassified *Cyclobacteriaceae*, *Lactococcus*, *Faecalibacterium*, unclassified *Chitinophagaceae*, *Bacteroides*, unclassified C111, unclassified *Comamonadaceae*, and *Geobacillus*. The relative abundance of *Cetobacterium* ranged from 30 to 42% and *Cupriavidus* ranged from 11.7 to 24% (Fig. 3b). Alpha diversity includes ACE, Chao1, Shannon and Simpson indexes, etc., which are often used to evaluate biodiversity. The results of this study show that the intestinal microbial diversity and abundance of wild fish are higher than those of cultured fish, but the difference is not significant (*P*-value > 0.05) (Table 3).

**Fig. 4:**
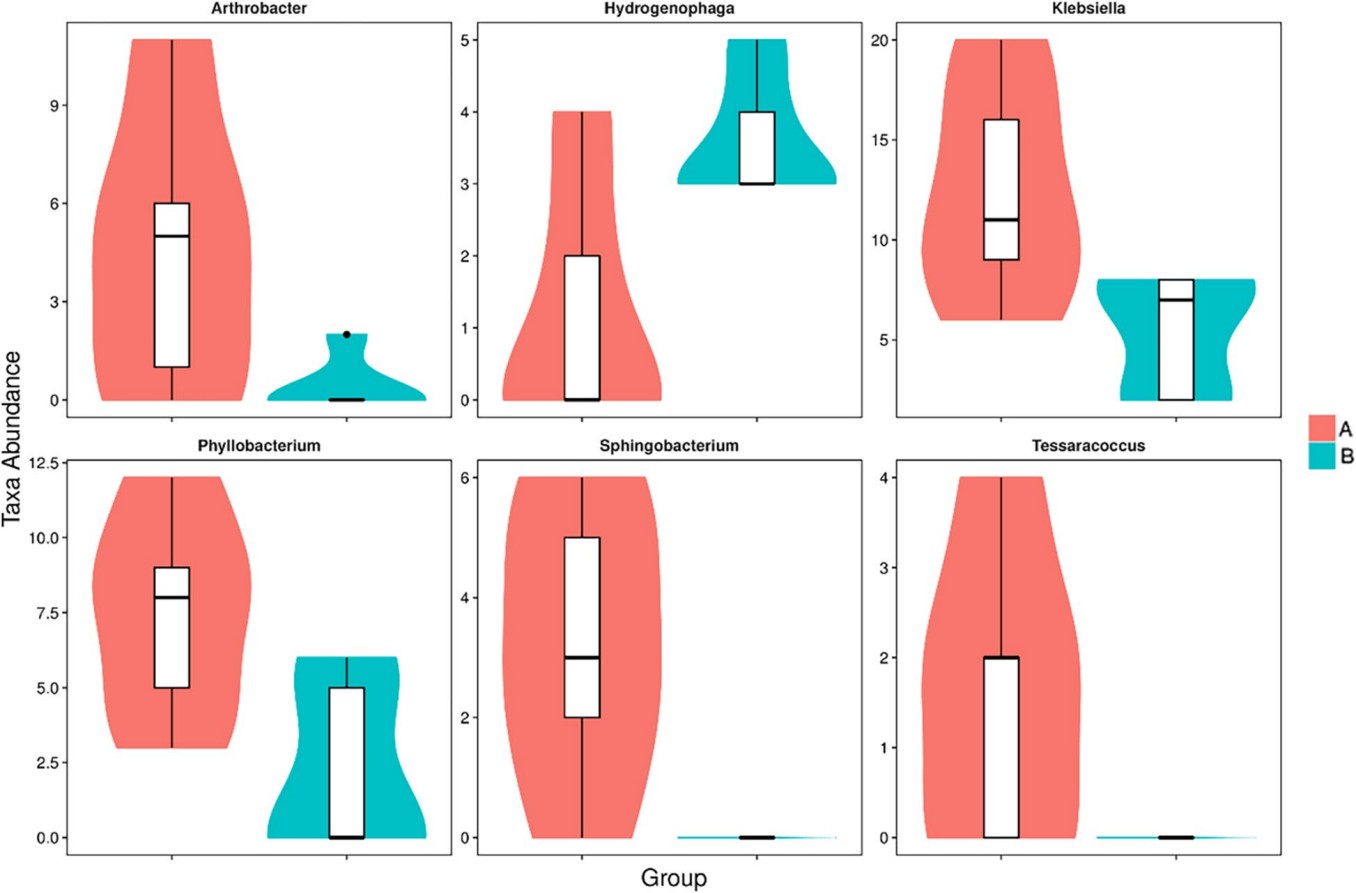
Metastats analysis of the intestinal microbiota in the wild and cultured *G. maculatum* gut microbiota, showing relative abundance of bacterial genera that differ significantly between wild and cultured *G. maculatum* gut microbiota

**Fig. 5:**
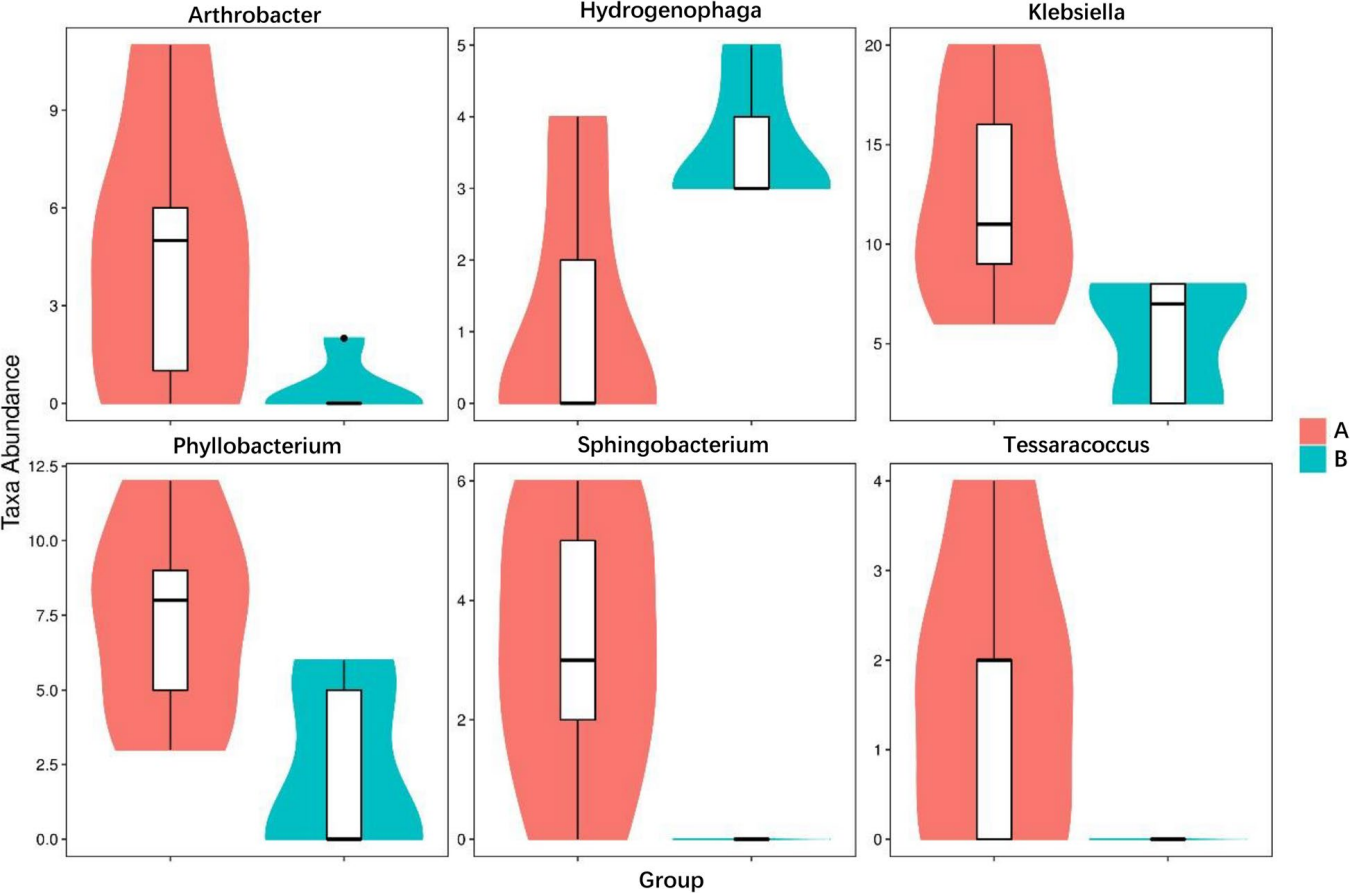
The function prediction of *G. maculatum* gut microbiota based on KEGG

Specific taxa that were differentially distributed between wild and cultured *G. maculatum* were identified using Metastats. The results are shown in Fig. 4, which depicts each genus showing a significant difference between the wild *G. maculatum* (group A) and cultured *G. maculatum* (group B) intestinal microbiota. At the genus level, a total of six genera were differentially represented between the two conditions. *Arthrobacter*, *Klebsiella*, *Phyllobacterium*, *Sphingobacterium* and *Tessaracoccus* were much higher in the wild *G. maculatum* than in the cultured *G. maculatum*, while *Hydrogenophaga* was higher in the cultured *G. maculatum*. The results of PCoA showed that the degree of dispersion within each sample group in group A and group B was small, while the degree of dispersion between groups was large (Fig. 5).

### Function prediction of *G. maculatum* intestinal microbiota

Phylogenetic Investigation of Communities by Reconstruction of Unobserved States (PICRUSt) analysis showed that metabolism, genetic information processing, and the environmental information processing are the crucial KEGG pathways in the microbiome community (Fig. 5). The results also show that the main functions of genes included genes from biodegradation and metabolism of xenobiotics, metabolism of cofactors and vitamin pathway, energy metabolism pathway, translation of genetic information processing pathway, carbohydrate metabolism pathway, amino acid metabolism and pathway of metabolism, replication and repair and genetic information processing pathway and membrane transport of environmental information processing.

## Discussion

Accumulating evidence shows that vertebrate intestinal microbiota can stimulate the development of host immune system, assist the host to obtain nutrients and resistance to opportunistic pathogens. Therefore, the intestinal microbiota plays an indispensable role in host health [19]. *G. maculatum* is an aquaculture species with important economic and ecological value that inhabits the Tibetan Plateau (4200 m above sea level). The unraveling of the structure of its intestinal microbiota and identification of its core microbiome as well as the intricate host-microbe symbioses and functions are essential to the ability to use the benefits of a healthy microbiome to the advantage in *G. maculatum* culture, and furthermore, to gain a deeper understanding of the roles of bacteria in vertebrate health to develop effective strategies for regulating intestinal microbiota to improve the health of *G. maculatum*. Recent technological advances in sequencing have led to a much more comprehensive assessment of the intestinal microbiota. 16S rRNA gene sequencing has been used widely in the study of the composition of the intestinal microbiota of multiple organisms, including freshwater fish and marine fish [20–23]. From the results of this studies, 16S rRNA gene sequencing has been found to be a very effective method for deep study of the intestinal microbiota composition and structure of *G. maculatum*.

The results revealed that the cultured and wild *G. maculatum* have high similarity at phylum and genus levels, which suggested that wild *G. maculatum* in their natural environment and *G. maculatum* that have been maintained in an artificial environment obtain a common intestinal microbiota. The results of 16S rRNA gene sequencing emphasized that a core microbiota are present in both *G. maculatum* intestinal microbiota. The concept of a core intestinal microbiota has been explored in the context of mammalian hosts [24, 25], rat [26], and bony fishes [23, 27]. In this study, the Chao1 value, Shannon and Simpson indexes of the wild *G. maculatum* intestinal microbiota were higher than those of cultured fish, indicating higher bacterial diversity in the wild *G. maculatum* gut microbiota compared to the cultured *G. maculatum* gut microbiota [28]. As an “extra organ”, the gut microbiota are involved in the metabolic, nutritional, physiological, and immunological processes of the host, to help its host to reshape their growth, health and development [29]. In the wild *G. maculatum* gut microbiota, higher bacterial diversity means that there are more microorganisms to help the wild *G. maculatum* cope with the sophisticated living conditions and increase nutrient absorption. For example, Metastats analysis showed that *Arthrobacter* was much higher in the wild *G. maculatum* than in the cultured *G. maculatum*. It is reported that Arthrobacter is a kind of probiotics commonly used in aquaculture. The metabolites of this kind of bacteria, such as amino acids, vitamins and enzymes, have high nutritional value and resist opportunistic pathogens [30–32].

The genus of *Cetobacterium* is common in intestinal tracts of various fish such as carp, channel catfish, Asian seabass, goldfish, zebrafish, rainbow trout, etc. [9, 33–38]. *Cetobacterium* was the most abundant genus without any significant differences in both wild and cultured samples of *G. maculatum*, suggesting that *Cetobacterium* spp. is a core species in the *G. maculatum* gut and plays an important role in the growth and development of *G. maculatum*. *Cetobacterium* play a vital role in the intestines of many herbivorous fishes because it is related to the breakdown of carbohydrates, thereby assisting the host in the absorption and utilization of nutrients [39]. As we all know, *Cetobacterium* produces a large amount of vitamin B12, which can inhibit the growth of potential pathogens [40] and promote protein biosynthesis. It acts as a growth factor in many fishes, and a lack of *Cetobacterium* usually affects the growth and development of the host [41].

In the gut of both wild and cultured samples of *G. maculatum*, there were large numbers of *Cupriavidus*, which is the biggest unique feature of *G. maculatum* microbiota. To date, there had been no such reports in other fish intestinal microbiota, so this aspect is the most interesting feature of *G. maculatum* intestinal microbiota. The genus *Cupriavidus* was described by Makkar and Casida in 1987 to accommodate species that are characterized as Gram-stain-negative, motile by 2 to 10 peritrichous flagella, oxidase positive, catalase positive, chemoheterotrophic, can use several amino acids but not L-lysine or L-methionine as the sole source of carbon and nitrogen. The most interesting character of this genus is that they are resistant to copper at concentrations up to at least 800 μM, and growth initiation is stimulated by copper [42]. Copper is an indispensable trace element for fish. Lack of dietary copper causes growth retardation and poor feed efficiency in carp, grouper, and yellow catfish [43]. Supplementing channel catfish diets with copper sulfate, which can also be used as a therapeutic in the water, significantly increased fish resistance to pathogens such as *Flavobacterium columnare*, which can cause the most commercially detrimental bacterial diseases of channel catfish [44]. However, at elevated concentrations (i.e., > 1–2 mM), copper becomes toxic for most cells [45]. The upper reaches of the Yarlung Zangbo River Basin have been proved to contain a large number of copper resources and other related resources [46], which led to the high content of Cu in the upper and middle reaches of the Yarlung Zangbo River [47]. High pH, high Cu content, and low temperature are adverse conditions unfavorable to the growth of fish. *G. maculatum* can survive under these conditions, so they must have the physiological characteristics to solve these adverse conditions. According to the results, there are large numbers of *Cupriavidus* in the gut microbiota of *G. maculatum*, which may play a vital role in the adaptation of *G. maculatum* to the water of the Yarlung Zangbo River with high Cu content. On the one hand, because *Cupriavidus* possesses a good ability for the absorption and removal of copper from the water, *Cupriavidus* can make *G. maculatum* able to live in the water with a relatively high concentration of copper ions. On the other hand, because there are many *Cupriavidus* in the gut microbiota of *G. maculatum*, this observation suggests that when we feed *G. maculatum* artificially, we can increase the content of copper ions in its feed diet, which can fulfill the demand of *G. maculatum* for copper ion and can also inhibit the growth of pathogenic bacteria, so that *G. maculatum* can grow healthily under the conditions of artificial culture. However, we need to verify through experiments what concentration of copper ion in the diet is appropriate. Therefore, future research could be focused on the relationship between *G. maculatum* and Cupriavidus. How does Cupriavidus affect the survival of *G. maculatum* in its high altitude, low temperature, high Cu-content habitat? How high a concentration of copper ion in the diet is appropriate?

## Conclusions

The results of this study demonstrated that the intestinal microbiota of wild and cultured *G. maculatum* are similar in phylum and genus level structure. In addition, cetobacterium is the main component of *G. maculatum*’s intestinal core microbiota, indicating that cetobacterium plays a vital role in *G. maculatum*’s adaptation to the water environment of the Yarlung Zangbo River with high copper content.

## Data Availability

The datasets used and analysed during the current study are available from the Genebank of NCBI, Sequence Read Archive (SRA) under access number: PRJNA769970.

## Abbreviations

*G. maculatum*: *Glyptosternum maculatum*
16S rRNA: 16S ribosomal RNA
OTUs: Operational Taxonomic Units
RDP: Ribosomal Database Project
ACE: Abundance-based Coverage Estimation
PCA: Principal Component Analysis
PCoA: Principal Coordinate Analysis
UPGMA: Unweighted Pair Group Method with Arithmetic mean
PICRUSt: Phylogenetic Investigation of Communities by Reconstruction of Unobserved States
KEGG: Kyoto Encyclopedia of Genes and Genomes
